# Parental Media Literacy and Children’s Epistemic Curiosity in Screen-Based Media Use: Cross-Sectional Survey Study

**DOI:** 10.2196/89929

**Published:** 2026-09-15

**Authors:** Yoojin Chung, Peter J Schulz

**Affiliations:** 1 Division of Communication and Media, Ewha Womans University, 52, Ewhayeodae-gil, Seodaemun-gu, Seoul, Seoul, 03760, Republic of Korea, 41 58-666-4724; 2 Faculty of Communication, Culture and Society, Università della Svizzera Italiana, Lugano, Switzerland

**Keywords:** epistemic curiosity, parental media literacy, family dynamics, parent-child interaction, screen-based media, digital childhood

## Abstract

**Background:**

Research on children’s media use has traditionally focused on risks and negative outcomes. However, screen-based media may also provide opportunities for learning and curiosity-driven exploration. As young children increasingly engage with digital media within family contexts, parental media literacy may represent an important competency associated with children’s media experiences and developmental outcomes.

**Objective:**

This study examined the associations between parental media literacy and children’s epistemic curiosity during screen-based media use. Specifically, it investigated how 4 dimensions of parental media literacy—technological use, critical understanding, self-expression, and norm compliance—were associated with 2 dimensions of children’s epistemic curiosity: interest-type and deprivation-type curiosity.

**Methods:**

Between July 31, 2024, and August 21, 2024, we conducted an online cross-sectional survey of 1020 parents of children aged 7 to 9 years in South Korea, including 340 parents in each age group. Household income, parental media literacy, and children’s epistemic curiosity were assessed using validated 5-point Likert scales. Separate structural equation models were estimated to examine the direct associations between parental media literacy and children’s epistemic curiosity and the indirect associations between household income and children’s epistemic curiosity through parental media literacy.

**Results:**

Technological use was positively associated with both interest-type (β=.32; *P*<.001) and deprivation-type (β=.18; *P*<.001) curiosity. Critical understanding, self-expression, and norm compliance were also positively associated with both interest-type (β=.20 to .26; *P*<.001 in all cases) and deprivation-type (β=.21 to .23; *P*<.001 in all cases) curiosity. Household income showed significant indirect associations with interest-type curiosity through all 4 dimensions of parental media literacy (β=.005 to .009; *P*<.05 in all cases), whereas indirect associations with deprivation-type curiosity were observed only through critical understanding (β=.007; *P*=.003), self-expression (β=.011; *P*<.001), and norm compliance (β=.004; *P*=.03).

**Conclusions:**

The findings suggest that parental media literacy may extend beyond an individual competency and be associated with children’s curiosity-driven engagement with digital media. By highlighting parental media literacy as a potentially modifiable factor, this study underscores the importance of supporting parents as active participants in children’s digital learning environments.

## Introduction

### Parental Media Literacy and Children’s Epistemic Curiosity in Screen Media Use

In the pervasive and rapidly evolving digital environment, parents play a pivotal role in shaping young children’s media use, bearing primary responsibility for guiding their engagement with digital media [1]. Children’s early exposure to digital technology is influenced by multiple family-level factors [2], including socioeconomic status (SES) and parental educational level [3], parents’ expectations of and attitudes toward children’s media use [4], and everyday patterns of family interaction with digital technologies [5]. These factors shape children’s media experiences and may affect their developmental and social outcomes. Although parental factors have been widely studied across environmental, social, and emotional domains, the interpersonal and dynamic aspects of parent-child interactions around media use—particularly those related to parental competencies—remain underexplored [1].

Media literacy refers to the ability to access, interpret, evaluate, and create media messages in contemporary digital environments [6]. Previous research suggests that media literacy can reduce negative media influences while supporting more positive and informed media experiences [7]. By fostering critical thinking, media literacy can encourage more reflective media practices and protective attitudes [8]. However, traditional media literacy studies primarily emphasize risk mitigation and self-referential effects (eg, parents’ own media efficacy), often neglecting the critical impact of parental media literacy on children’s positive media experiences and cognitive development. Unlike parental mediation, which refers to specific behavioral strategies used to manage children’s media use, parental media literacy reflects a broader competency encompassing how parents access, understand, evaluate, and engage with media.

Rather than examining specific parental mediation behaviors, this study conceptualized parental media literacy as a broader parenting competency and investigated how it relates to children’s epistemic curiosity during screen-based media use. Epistemic curiosity refers to a desire for new information that motivates knowledge acquisition and exploratory behavior [9]. In digital environments where children encounter vast amounts of information, epistemic curiosity may support learning, problem-solving, and meaningful engagement with media content [10,11].

This study was conducted in South Korea, a highly digitalized society where children are exposed to digital media from an early age and where parents face increasing responsibility for guiding children’s media use. This issue may be particularly salient in South Korea, where digital media adoption is widespread and parents are increasingly expected to support children’s media experiences. However, many contemporary parents grew up without formal media literacy education and must navigate rapidly evolving digital environments while helping their children engage with media. Examining parental media literacy in this context provides valuable insights into how family-level competencies relate to children’s positive media experiences and learning-related outcomes in everyday digital environments.

### Media Literacy: Evolution and Outcomes

Media literacy originally referred to the knowledge and skills that enable individuals to understand media critically and use it appropriately [12,13]. Early conceptualizations emphasized the ability to access, analyze, evaluate, and create media messages in various forms [14]. As media environments have become more complex, the scope of media literacy has expanded beyond protection from harmful content to include competencies related to participation, privacy management, information evaluation, self-expression, ethical engagement, and the ability to maximize opportunities while minimizing risks in digital media use [7]. Although the specific dimensions of media literacy continue to evolve alongside technological change, scholars broadly agree that media literacy supports more informed, responsible, and effective media use.

Most research on media literacy has focused on its effects at the individual level. A substantial body of literature has demonstrated that media literacy is associated with greater critical thinking, improved understanding of media messages, stronger awareness of persuasive intent, and healthier media-related attitudes and behaviors [8,15,16]. However, media use rarely occurs in isolation. Individuals engage with media within social environments where interactions with family members, peers, and other social actors shape how media content is interpreted and experienced. Consequently, media literacy may extend beyond an individual competency and function as a form of social influence, affecting not only the person who possesses it but also those with whom they interact.

Although this possibility has received limited empirical attention, there are theoretical reasons to expect that media literacy may have implications beyond the individual. Media experiences are embedded within interpersonal relationships, where people exchange information, discuss media content, model behaviors, and influence one another’s interpretations of media messages. Consequently, media literacy may function not only as an individual competency but also as a form of social influence.

This possibility may be particularly relevant in family settings, where parents play a central role in structuring children’s media environments and guiding their engagement with digital media. Parent-child relationships are characterized by frequent interaction and asymmetries in knowledge and experience, making them a likely context in which media-related competencies may extend beyond the individual. Young children rely heavily on their parents to interpret media content, navigate digital environments, and make decisions about media use. Examining parental media literacy, therefore, provides an opportunity to explore how media-related competencies may shape children’s media experiences and learning-related outcomes.

### Parental Media Literacy as a Parenting Competency

Parents play a central role in shaping children’s media experiences. Existing research has primarily examined this influence through parental mediation strategies, such as restrictive mediation, explanatory mediation, and coviewing [17,18]. However, parents’ influence extends beyond observable mediation behaviors. Parents shape children’s media experiences through the media environments they create, the beliefs and attitudes they communicate, the resources and technologies they provide, and the ways in which they themselves engage with media [19].

For contemporary parents, these influences are particularly important in digital environments. Many parents grew up during a period of rapid technological change and did not receive formal media literacy education. Instead, they developed their understandings, habits, and beliefs through direct experiences with emerging media technologies. Paradoxically, these same individuals are now expected to guide their children’s media use and help them navigate increasingly complex digital environments. As a result, parents vary substantially in their ability to critically evaluate media, support children’s engagement with digital content, and foster constructive media experiences. In this study, these competencies were conceptualized as parental media literacy.

SES may further shape the development of parental media literacy. Parents with greater educational and economic resources often have broader access to digital technologies, information resources, and learning opportunities, which may facilitate the acquisition of media-related knowledge and skills [20-22]. Higher-SES families may also be more likely to adopt and engage with emerging technologies, providing parents with greater opportunities to develop familiarity with and competence in digital environments. Consequently, SES may influence children’s media experiences not only through material resources but also through its association with parents’ media literacy. Examining SES alongside parental media literacy, therefore, provides a more comprehensive understanding of the family factors associated with children’s media engagement and learning-related outcomes.

Much of the existing literature on children’s media use has focused on risks and problematic outcomes, reflecting concerns about excessive screen time, problematic use, and exposure to harmful content. Although these concerns remain important, digital media has become an unavoidable part of children’s everyday lives. Rather than viewing media solely as a source of risk, it is equally important to understand the conditions under which children experience positive and developmentally beneficial outcomes. One such outcome may be epistemic curiosity, a desire for knowledge and understanding that motivates learning and exploration. Examining whether parental media literacy is associated with children’s epistemic curiosity may therefore provide insights into how family environments support constructive engagement with digital media.

### Epistemic Curiosity in Digital Media Environments

Epistemic curiosity refers to a desire for knowledge and understanding that motivates information seeking, exploration, and learning [9]. Because it encourages children to engage with new ideas, ask questions, and resolve uncertainty, epistemic curiosity is considered an important developmental outcome linked to cognitive growth and learning [23-25].

Prior research distinguishes between 2 dimensions of epistemic curiosity: interest-type and deprivation-type curiosity [26]. Interest-type curiosity reflects enjoyment of novelty and discovery, whereas deprivation-type curiosity arises from a desire to resolve gaps in knowledge or understanding [27,28]. Although these dimensions differ in motivation, both are associated with active information seeking and intellectual exploration [26,29,30].

The development of epistemic curiosity begins early in life, when children are actively constructing knowledge about the world around them. Prior research suggests that curiosity can be fostered through exposure to novel information, opportunities for exploration, and encounters with uncertainty that motivate information seeking [31-34]. Because epistemic curiosity supports learning, problem-solving, and knowledge acquisition, it is increasingly recognized as an important developmental resource during childhood.

Epistemic curiosity may be particularly relevant in contemporary digital environments. Children are routinely exposed to novel information, diverse perspectives, and new learning opportunities through screen-based media. As digital media increasingly serves as a gateway through which children encounter and learn about the world, media experiences may play an important role in stimulating curiosity and exploration. While much research has focused on the risks of digital media use, these environments may also provide opportunities for curiosity-driven learning and intellectual engagement. Given parents’ central role in shaping children’s media experiences, parental media literacy may be associated with how children engage with these opportunities and experience epistemic curiosity during media use.

### The Current Study

On the basis of the theoretical arguments and empirical gaps discussed above, this study examined the relationships among parental media literacy, SES, and children’s epistemic curiosity, including both interest type and deprivation type. Specifically, this study investigated whether parental media literacy was associated with children’s epistemic curiosity during screen-based media use and whether parental media literacy may help explain the relationship between SES and children’s epistemic curiosity.

**Figure 1.**
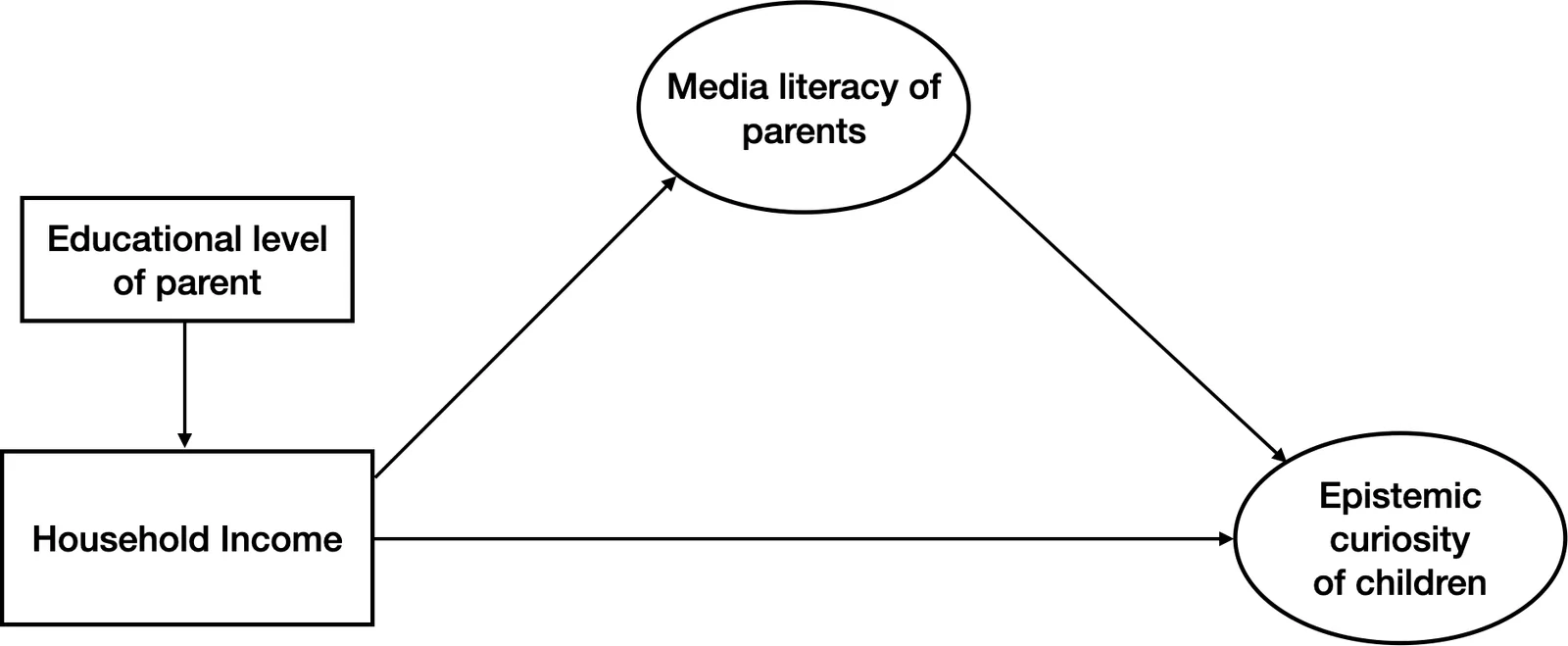
Research model.

Figure 1 shows the conceptual model linking household income, parental media literacy, and children’s epistemic curiosity. On the basis of prior literature, parental media literacy is expected to be positively associated with children’s epistemic curiosity, and SES is expected to be associated with both parental media literacy and children’s epistemic curiosity. The following hypotheses were proposed:

- *Hypothesis 1*—higher levels of parental media literacy (technological use, critical understanding, self-expression, and norm compliance) will be positively associated with children’s epistemic curiosity (interest type and deprivation type) during screen-based media use.
- *Hypothesis 2*—higher SES will be positively associated with children’s epistemic curiosity during screen-based media use.
- *Hypothesis 3*—parental media literacy will mediate the association between SES and children’s epistemic curiosity during screen-based media use.

## Methods

### Participants and Procedure

Data were collected from a target population of 1020 primary caregiver parents of children aged 7 to 9 years in South Korea via a professional online research panel. The survey was administered between July 31, 2024, and August 21, 2024, and included 340 parents of children in each age group (7, 8, and 9 years). Most respondents (n=887, 87%) were mothers, and 13.0% (n*=*133) were fathers. The survey, designed for quantitative analysis, commenced with a screening question to confirm the respondent’s status as the primary caregiver and included detailed measures regarding the child’s media exposure, device access, use patterns, and the control variables used in the final analysis. All items were measured on a 5-point Likert scale except SES, which was measured categorically.

### Ethical Considerations

This study received ethics approval from the Ewha Womans University Institutional Review Board (protocol number ewha-202103-0028-04). Participants provided informed consent through the online platform.

### Measurements

All constructs were assessed using previously validated scales. Confirmatory factor analyses (CFAs) were conducted to evaluate the measurement properties of the scales. Table 1 shows the CFA results, reliability estimates, and validity indicators for all study variables.

**Table 1.** Measurement properties of the study variables^a^.

Construct	Mean (SD)	Loading range	McDonald ω	AVE^b^
TU^c^	4.31 (0.52)	0.63-0.83	0.84	0.51
CU^d^	3.95 (0.56)	0.31-0.87	0.75	0.44
SE^e^	3.80 (0.69)	0.56-0.76	0.83	0.46
NC^f^	4.14 (0.54)	0.35-0.88	0.68	0.37
Interest-type curiosity	3.77 (0.55)	0.65-0.76	0.80	0.50
Deprivation-type curiosity	3.36 (0.65)	0.70-0.83	0.84	0.56

a The 4-factor confirmatory factor analysis provided partial support for the proposed multidimensional structure (comparative fit index [CFI]=0.87; Tucker-Lewis index [TLI]=0.85; root mean squared error of approximation [RMSEA]=0.07; standardized root mean squared residual [SRMR]=0.07), whereas the 2-factor model for epistemic curiosity demonstrated good fit (CFI=0.97; TLI=0.96; RMSEA=0.06; SRMR=0.04). Discriminant validity was considered acceptable because the squared latent correlations were lower than the average variance extracted estimates for most constructs.

b AVE: average variance extracted.

c TU: technological use.

d CU: critical understanding.

e SE: self-expression.

f NC: norm compliance.

For parental media literacy, a 4-factor model demonstrated modest fit (*χ*^2^_146_=959.8; comparative fit index [CFI]=0.87; root mean squared error of approximation [RMSEA]=0.07; standardized root mean squared residual [SRMR]=0.07). For epistemic curiosity, a 2-factor model demonstrated good fit (*χ*^2^_19_=85.0; CFI=0.97; RMSEA=0.06; SRMR=0.04). Standardized factor loadings ranged from 0.31 to 0.88 for parental media literacy and from 0.65 to 0.83 for epistemic curiosity. Internal consistency was assessed using the McDonald ω.

### Epistemic Curiosity

Epistemic curiosity during screen-based media use (parent reported) was measured using a 10-item scale adapted from the work by Piotrowski et al [25] that assesses children’s curiosity through caregiver observation. The scale was previously translated into Korean and validated [29].

The scale consists of 2 dimensions: intellectual interest (5 items; mean 3.77, SD 0.55; ω*=*0.80) and informational deprivation (5 items; mean 3.36, SD 0.65; ω=0.84). A 2-factor CFA supported the distinction between interest- and deprivation-type curiosity. Standardized factor loadings ranged from 0.65 to 0.76 for interest-type curiosity and from 0.70 to 0.83 for deprivation-type curiosity. Items were measured on a 5-point Likert scale ranging from 1 (“strongly disagree”) to 5 (“strongly agree”).

### Parental Media Literacy

Parental media literacy was measured using a validated multidimensional scale developed by Ahn et al [35]. On the basis of their established relevance to child outcomes and parent-child mediation effectiveness, 4 key dimensions were selected for this study: technological use (5 items; mean 4.31, SD 0.52; ω=0.84), critical understanding (4 items; mean 3.95, SD 0.56; ω=0.75), self-expression (5 items; mean 3.80, SD 0.69; ω=0.83), and norm compliance (4 items; mean 4.14, SD 0.54; ω=0.68). The reliability coefficient for norm compliance was somewhat lower than those observed for the other dimensions. However, the value remained within the range often considered acceptable for established multidimensional scales in social science research, particularly when constructs encompass heterogeneous behavioral indicators. Accordingly, the scale was retained to preserve the theoretical breadth of the parental media literacy framework.

A 4-factor CFA generally supported the multidimensional structure of parental media literacy. Standardized factor loadings ranged from 0.63 to 0.83 for technological use, 0.31 to 0.87 for critical understanding, 0.56 to 0.76 for self-expression, and 0.35 to 0.88 for norm compliance. Items were measured on a 5-point Likert scale ranging from 1 (“strongly disagree”) to 5 (“strongly agree”).

### Household SES and Control Variables

SES was operationalized using 2 parent-reported measures. Household income, the primary SES proxy for the main hypothesis (hypotheses 2 and 3), was measured using a 10-point categorical scale (from less than KRW 2.5 million to more than KRW 10.5 million, with intervals of KRW 1 million [KRW 1=US $0.00072 as of August 20, 2026]). For the analysis, this categorical variable was treated as a continuous ordinal variable. Parental educational level, categorized into 5 options (eg, high school graduate and bachelor’s degree), was measured to serve as a control variable in the analysis. The gender of the primary caregiver (mother=0; father=1) was also included as a control variable.

### Analysis Plan

Prior to hypothesis testing, the psychometric properties of all latent variables were rigorously assessed through CFA. Through the validated measurement models established via CFA, this study used structural equation modeling via the *lavaan* package in R (R Foundation for Statistical Computing) [36] to test the hypothesized relationships, including the mediation effect (hypothesis 3).

Because parental media literacy was conceptualized as 4 distinct dimensions and epistemic curiosity was conceptualized as 2 theoretically distinct outcomes (interest and deprivation type), separate structural equation models were estimated for each combination of media literacy and curiosity dimensions (4 × 2 = 8 models). This approach allowed the associations between specific dimensions of parental media literacy and specific dimensions of epistemic curiosity to be examined independently, consistent with the study’s aim of understanding the role of each media literacy dimension rather than evaluating their relative effects within a single model.

Given the observed nonnormal distribution of the variables and the categorical nature of SES, indirect effects were tested using 5000 bias-corrected bootstrap samples, following the recommendations of Preacher and Hayes [37].

Parental educational level was included as a covariate in all structural equation models. This decision was made because the educational level was observed to influence both household income and parental media literacy and the sample exhibited a skew toward highly educated parents. The primary caregiver’s gender was also included as a covariate in all models.

## Results

### Descriptive Statistics

A total of 1020 completed questionnaires were collected and statistically analyzed. The age range for participating parents was 27 to 56 years. Mothers’ ages ranged from 27 to 51 years (mean 40.37, SD 3.72 years), and fathers’ ages ranged from 27 to 56 years (mean 41.41, SD 3.97 years). The sample exhibited a higher educational attainment compared to the national average (41% with higher education), with 92.5% (n=944) having an associate college degree or higher. Specifically, 21.8% (n=222) had an associate college degree, 57.5% (n*=*586) had a bachelor’s degree, and 13.3% (n=136) had a graduate degree or higher. Only 0.2% (n=2) had not completed middle school, and 7.3% (n=74) were high school graduates. The sample also reported relatively high household incomes (mean KRW 5.5 million to 6.5 million, SD KRW 2.13 million). The distribution was near normal (skewness=0.25; kurtosis=–0.62), with 46.3% (n=472) reporting a household income exceeding KRW 6.5 million.

Correlations between all study variables were examined and are shown in Table 2. Findings indicated statistically significant correlations among most variables except between parental educational level and 2 dimensions of media literacy: technological use and norm compliance.

**Table 2.** Correlation between study variables (N=1020).

	Interest type	Deprivation type	TU^a^	CU^b^	SE^c^	NC^d^	Income	Educational level
Deprivation type	0.52^e^	1	0.14^e^	0.20^e^	0.26^e^	0.19^e^	0.11^e^	0.10^f^
TU	0.31^e^	0.14^e^	1	0.48^e^	0.49^e^	0.42^e^	0.08^f^	–0.04
CU	0.26^e^	0.20^e^	0.48^e^	1	0.42^e^	0.37^e^	0.11^e^	0.13^e^
SE	0.26^e^	0.26^e^	0.49^e^	0.42^e^	1	0.36^e^	0.14^e^	0.10^f^
NC	0.26^e^	0.19^e^	0.42^e^	0.37^e^	0.36^e^	1	0.08^f^	0.03
Income	0.11^e^	0.11^e^	0.08^f^	0.11^e^	0.14^e^	0.08^f^	1	0.26
Educational level	0.06^f^	0.10^f^	–0.04	0.13^e^	0.10^f^	0.03	0.26^e^	1

a TU: technological use.

b CU: critical understanding.

c SE: self-expression.

d NC: norm compliance.

e *P*<.001.

f *P*<.05.

### Parental Media Literacy and Children’s Epistemic Curiosity

The results of the structural equation models with interest-type epistemic curiosity as the dependent variable are shown in Table 3. Model fit indexes for interest-type epistemic curiosity as the dependent variable indicated acceptable fit across all dimensions of parental media literacy (technological use: *χ*^2^_2_=7.5, *P*<.001, CFI=0.97, RMSEA=0.05, and SRMR=0.02; critical understanding: *χ*^2^_2_=11.9, *P*<.001, CFI=0.94, RMSEA=0.07, and SRMR=0.03; self-expression: *χ*^2^_2_=4.8, *P*=.09, CFI=0.98, RMSEA=0.04, and SRMR=0.02; norm compliance: *χ*^2^_2_=1.4, *P*=.50, CFI=1.00, RMSEA=0.00, and SRMR=0.00). All structural equation models were estimated under the same analytical framework and included the same set of covariates. The models were overidentified (df*=*2), allowing model fit to be evaluated using conventional fit indexes.

**Table 3.** Interest-type curiosity results.

PML^a^ dimension	Income→PML, β	PML→interest-type curiosity, β	Income→interest-type curiosity, β	Indirect effect, β
TU^b^	.02^c^	.32^d^	.02^d^	.006^c^
CU^e^	.03^d^	.24^d^	.02^c^	.007^c^
SE^f^	.05^d^	.20^d^	.02^c^	.009^d^
NC^g^	.02^c^	.26^d^	.02^d^	.005^c^

a PML: parental media literacy.

b TU: technological use.

c *P*<.05

d *P*<.001.

e CU: critical understanding.

f SE: self-expression.

g NC: norm compliance.

Consistent with hypothesis 2, the direct effect of household income on children’s interest-type epistemic curiosity was statistically significant across all 4 dimensions of parental media literacy (β*=*.02; *P*<.05). This finding suggests that higher household income is consistently associated with greater experience of interest-type epistemic curiosity in children during media use.

Regarding the mediation hypothesis, the indirect effect of parental media literacy significantly mediated the relationship between household income and interest-type epistemic curiosity across all 4 parental media literacy dimensions, supporting hypothesis 3 for interest-type curiosity. The standardized indirect effects were significant through technological use (β=.006; *P=*.02), critical understanding (β*=*.007; *P*=.003), self-expression (β*=*.009; *P<*.001), and norm compliance (β*=*.005; *P=*.005). Although statistically significant, the indirect effects were small in magnitude.

The results of the structural equation model with deprivation-type epistemic curiosity as the dependent variable are shown in Table 4. The model fit indexes for all 4 models suggested an adequate fit (technological use: *χ*^2^_2_=14.0, *P*<.001, CFI=0.91, RMSEA=0.07, and SRMR=0.04; critical understanding: *χ*^2^_2_=15.0, *P*<.001, CFI=0.91, RMSEA=0.08, and SRMR=0.04; self-expression: *χ*^2^_2_=8.2, *P*=.02, CFI=0.96, RMSEA=0.06, and SRMR=0.03; norm compliance: *χ*^2^_2_=5.8, *P*=.06, CFI=0.97, RMSEA=0.04, and SRMR=0.02).

Regarding the direct effect of household income (hypothesis 2), the relationship with deprivation-type epistemic curiosity was statistically significant for 3 dimensions of parental media literacy: critical understanding (β*=*.03; *P=*.005), self-expression (β*=*.02; *P=*.01), and norm compliance (β*=*.02; *P=*.002). However, the direct effect was not significant for the technological use dimension (β*=*–.00; *P=*.79). This finding partially supports hypothesis 2.

Regarding the mediation hypothesis (hypothesis 3), the indirect effect of household income on deprivation-type epistemic curiosity mediated by parental media literacy was significant for critical understanding (β*=*.007; *P=*.003), self-expression (β*=*.011; *P*<.001), and norm compliance (β*=*.004; *P=*.03). In contrast, the indirect effect through technological use was not statistically significant (β*=*.00; *P=*.74). This finding partially supports hypothesis 3 concerning deprivation-type curiosity. Although these indirect effects were statistically significant, their magnitudes were small and should be interpreted with caution.

**Table 4.** Deprivation-type curiosity results.

PML^a^ dimension	Income→PML, β	PML→deprivation-type curiosity, β	Income→deprivation-type curiosity, β	Indirect effect, β
TU^b^	.001	.18^c^	.001	.000
CU^d^	.03^c^	.21^c^	.03^e^	.007^e^
SE^f^	.05^c^	.23^c^	.02^e^	.011^c^
NC^g^	.02^e^	.22^c^	.02^e^	.004^e^

a PML: parental media literacy.

b TU: technological use.

c *P*<.001.

d CU: critical understanding.

e *P*<.05

f SE: self-expression.

g NC: norm compliance.

## Discussion

### Principal Findings

This study examined the role of parental media literacy in children’s epistemic curiosity during screen-based media use. In contemporary digital environments, children are routinely exposed to diverse information and learning opportunities through screen-based media, making it increasingly important to understand the factors that shape how they engage with these experiences. Across the 4 dimensions of parental media literacy, higher levels were generally associated with greater epistemic curiosity among children during media use. Although the magnitude of the associations varied across dimensions, the findings suggest that parental media literacy may be linked to children’s curiosity-driven engagement with digital media. Among the 4 dimensions, technological use showed a distinct pattern: it was directly associated with both interest- and deprivation-type curiosity, but the indirect association between household income and deprivation-type curiosity through technological use was not statistically significant.

### Parental Media Literacy and Children’s Epistemic Curiosity

Critical understanding, self-expression, and norm compliance were positively associated with both interest- and deprivation-type curiosity. Technological use was also positively associated with both forms of curiosity, although its association with deprivation-type curiosity was relatively weaker than that observed for interest-type curiosity.

One notable finding was that technological use showed a different pattern from those of the other dimensions of parental media literacy. While critical understanding, self-expression, and norm compliance demonstrated significant indirect associations linking household income to deprivation-type curiosity, technological use did not. Although technological use was directly associated with both interest- and deprivation-type curiosity, its indirect association between household income and deprivation-type curiosity was not statistically significant.

Technological use may increase children’s opportunities to encounter novel content and diverse information, thereby stimulating interest-based exploration (interest-type curiosity). One possible explanation is that deprivation-type curiosity, which reflects a desire to resolve uncertainty and knowledge gaps, may depend more heavily on higher-order competencies such as critical understanding, self-expression, and reflective engagement with media.

This distinct pattern warrants further investigation. Future research should examine why technological media skills appear to support children’s curiosity directly but do not function as a significant pathway linking household income to deprivation-type curiosity.

### Parental Media Literacy as a Pathway Linking SES and Epistemic Curiosity

The findings also suggest that parental media literacy may represent one potential pathway through which socioeconomic resources are associated with children’s media-related experiences. Household income was positively associated with parental media literacy, and indirect effects emerged across several dimensions of epistemic curiosity. These findings are consistent with those of previous research showing that families with greater socioeconomic resources often have increased access to educational opportunities and digital technologies that may support the development of media-related competencies.

The association between household income and parental media literacy may be particularly understandable for dimensions such as technological use, which often requires access to digital devices, platforms, and opportunities for hands-on engagement with emerging technologies. In contrast, competencies such as critical understanding, self-expression, and norm compliance may be more amenable to educational interventions and learning opportunities.

Importantly, unlike socioeconomic resources, parental media literacy represents a potentially modifiable competency. Although the present findings do not imply that media literacy can eliminate socioeconomic disparities, they suggest that parental media literacy may be one factor associated with children’s positive engagement with digital media regardless of family income. From this perspective, media literacy education may represent a promising avenue for supporting families as they navigate increasingly complex digital environments.

Although the indirect associations were statistically significant, their effect sizes were small in magnitude. Consequently, parental media literacy should be viewed as one of several factors that may be associated with the relationship between socioeconomic resources and children’s epistemic curiosity.

### Implications for Media Literacy Education

The present findings highlight the potential value of parental media literacy education. Contemporary parents are increasingly expected to guide children’s media use, yet many developed their own media practices through personal experience rather than formal media literacy training. As digital environments continue to evolve, parents face growing challenges in helping children navigate complex media ecosystems. The present findings suggest that parental media literacy may be associated with children’s curiosity-driven engagement with media, underscoring the importance of supporting parents as active participants in children’s digital learning environments.

Notably, parental media literacy represents a competency that can potentially be strengthened through education and intervention. Although media literacy education cannot eliminate broader socioeconomic inequalities, it may provide parents with practical tools to support children’s engagement with digital media. Educational programs that promote critical understanding, self-expression, and responsible media use may help parents create media experiences that encourage exploration, learning, and curiosity.

Given the importance of epistemic curiosity for children’s learning and development, future research should continue to explore how parental media literacy education may support positive media experiences and learning-related outcomes in childhood.

### Limitations

This study has several limitations. First, the data were cross-sectional, preventing conclusions regarding the direction of the associations observed. Although the findings suggest that parental media literacy is associated with children’s epistemic curiosity during screen-based media use, future longitudinal research is needed to better understand how these relationships develop over time.

Second, children’s epistemic curiosity was assessed through parent reports rather than direct observations or child self-reports. Although parent reports are commonly used when studying young children, they may not fully capture children’s subjective experiences during media use. In addition, because both predictor and outcome variables were obtained from the same respondent, shared method variance may have inflated some of the observed associations. Future research should incorporate multiple sources of data, including child reports, behavioral measures, observational approaches, and longitudinal designs.

Third, this study was conducted in South Korea, a highly digitalized society characterized by widespread media use and strong parental involvement in children’s education. In addition, participants were recruited through an online panel, which may have resulted in a sample with relatively high levels of parental engagement. Furthermore, mothers constituted most respondents. Although mothers often serve as primary caregivers for children in the target age group for this study, future research would benefit from including a more balanced representation of fathers to examine potential differences in parental media literacy and children’s media experiences.

### Conclusions

In conclusion, this study contributes to the growing literature on media literacy by examining parental media literacy in relation to children’s epistemic curiosity during screen-based media use. The findings suggest that parental media literacy may extend beyond an individual competency and be associated with children’s media-related experiences within family contexts. By highlighting parental media literacy as a potentially modifiable factor linked to children’s curiosity-driven engagement with digital media, this study underscores the importance of supporting parents as they navigate increasingly complex media environments. Future research should continue to explore how parental media literacy can be fostered and how it may contribute to positive media experiences and learning-related outcomes in childhood.

## Abbreviations

CFA: confirmatory factor analysis
CFI: comparative fit index
RMSEA: root mean squared error of approximation
SES: socioeconomic status
SRMR: standardized root mean squared residual
